# Oxidative Stress Score as a Simplified Surrogate for Prognostic Stratification and Therapeutic Decision-Making in Multiple Myeloma

**DOI:** 10.3390/ph18060878

**Published:** 2025-06-12

**Authors:** Qi Liang, Limei Zhang, Qianqian Huang, Weiran Lv, Zhijian Liang, Shutong Liu, Runcong Nie, Zhongjun Xia, Yang Liang, Yun Wang

**Affiliations:** 1 Department of Hematologic Oncology, State Key Laboratory of Oncology in South China, Sun Yat-Sen University Cancer Center, Guangzhou 510060, China; 2 Department of Hematology, First Affiliated Hospital of Sun Yat-Sen University, Guangzhou 510080, China

**Keywords:** newly diagnosed multiple myeloma, oxidative stress, overall survival, progression-free survival, lactate dehydrogenase, albumin, gamma-glutamyl transpeptidase

## Abstract

**Background:** Oxidative stress contributes to the initiation and progression of multiple myeloma and can be reflected by various biochemical indicators. However, whether systemic oxidative stress level can serve as a simple and effective alternative for prognostic risk stratification in newly diagnosed multiple myeloma (NDMM) patients remains to be explored and validated in large clinical cohorts. **Methods:** A retrospective analysis was conducted on 1107 NDMM patients, 774 patients were randomly assigned to the training cohort and 333 to the validation cohort. They were divided into two groups based on the oxidative stress score (OSS), calculated by the weights of systemic oxidative stress indicators. The relationship between systemic oxidative stress levels of multiple myeloma and prognosis were analyzed both in the training and validation cohorts. **Results:** Constructed by three oxidative stress-related indicators, OSS was associated with significantly shorter overall and progression-free survival in the high OSS group compared to the low OSS group. The 5-year AUC value of the time-dependent ROC for OSS was comparable to that of RISS, and significantly higher than that of the DS staging system. Moreover, patients with high OSS did not benefit significantly from standard combination therapy with IMiDs and PIs over monotherapy in terms of prognosis when compared to the low OSS group (all *p* < 0.05). **Conclusions:** OSS was observed to be an independent prognostic factor for overall survival and progression-free survival in patients with NDMM, suggesting systemic oxidative stress could serve as a new approach for accurate prognostic prediction and guiding treatment in multiple myeloma.

## 1. Introduction

Multiple myeloma (MM) is characterized by the aberrant build-up of clonal plasma cells in the bone marrow, leading to hypercalcemia, poor renal function, anemia, and bone destruction, accounting for approximately 10% of all hematological malignancies [1]. Globally, the incidence of MM increased by 126% from 1990 to 2016, and the age-standardized rate was 1.78 per 100,000 people in 2022 worldwide [2,3]. Autologous stem cell transplantation (ASCT) and novel immune treatments have transformed the natural course of the disease, resulting in significantly extended survival periods [4,5].

Despite therapeutic advances, clinical outcomes in MM remain heterogeneous due to variable tumor biology, patient characteristics, and treatment response. Among patients treated with similar first-line regimens based on immunomodulatory drugs (IMiDs) and proteasome inhibitors (PIs), median progression-free survival (PFS) and overall survival (OS) reach approximately 26 and 143 months, respectively, yet individual outcomes remain highly heterogeneous, spanning from a few months to beyond a decade [6,7]. These observations underscore the need for novel and broadly applicable prognostic models to identify patients with inferior treatment response and enhance risk stratification in MM.

Oxidative stress is a phenomenon arising from the imbalance in the production and accumulation of reactive oxygen species (ROS) in cells and tissues, and certain biochemical indicators can reflect oxidative stress status [8]. In MM, malignant plasma cells exhibit increased glycolytic activity and elevated ROS levels, while patients often display reduced systemic antioxidant capacity [9].

Previous studies have shown that lactate dehydrogenase (LDH) may promote the production of hydrogen peroxide (H_2_O_2_) and oxidative stress in cancer cells, both in vitro and in vivo [10]. Additionally, LDH serves not only as a marker but also as a mediator of oxidative stress and inflammation in animal models [11]. As an enzyme involved in the extracellular degradation of glutathione (GSH), gamma-glutamyl transferase (GGT) is considered an indicator of oxidative stress in various cancers. A rise in GGT activity represents elevated oxidative stress and antioxidant deficiency [12,13,14]. Albumin (ALB) is one of the major antioxidants in plasma, contributing to the regulation of reactive oxygen species metabolism and serving as an indicator of oxidative stress in various pathological conditions [15,16,17]. Creatine (CRE), C-reactive protein (CRP), blood urea nitrogen (BUN), and alkaline phosphatase (ALP) levels are elevated under oxidative stress in both animal models and critically ill patients but can be reduced by antioxidant treatment [18,19,20], suggesting that these biochemical indicators could be signs of systemic oxidative stress throughout the body.

Several staging systems have been developed to predict prognosis and facilitate the risk stratification of patients with MM, like Durie–Salmon (DS) staging, international staging systems (ISS) staging, revised ISS (RISS), and second revision of the ISS (R2ISS) staging [21,22]. Nevertheless, heterogeneity still exists in the prognosis of patients with newly diagnosed multiple myeloma (NDMM) within the same staging subgroup and advanced cytogenetic testing remains challenging in resource-constrained settings.

Therefore, this study aimed to establish a novel oxidative stress score (OSS) model based on the biochemical indicators of systemic oxidative stress to provide a new approach for the accurate prognostic prediction and support therapeutic decision-making in patients with NDMM.

## 2. Results

### 2.1. Model Construction and Analysis

**Figure 1:**
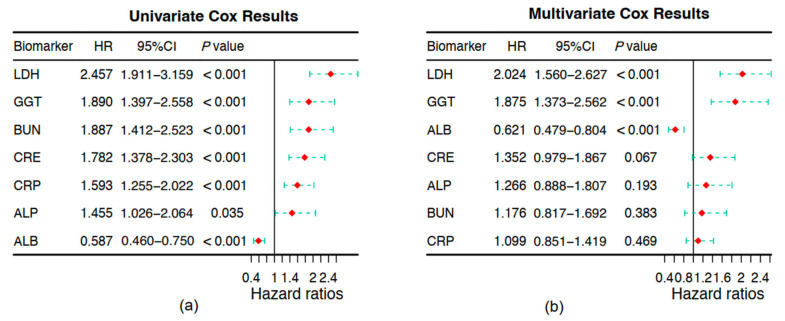
Construction of oxidative stress score (OSS). (**a**) Univariate Cox regression analysis of candidate oxidative stress-related biomarkers for overall survival. (**b**) Multivariate Cox regression analysis identifying the final variables included in the OSS model. Hazard ratios (HRs), 95% confidence intervals (CIs), and *p* values are shown.

Univariate and multivariate Cox regression analyses were conducted on 774 patients in the training cohort to identify independent prognostic factors and develop the OSS for NDMM. In univariate analysis, LDH (HR, 2.457, 95% CI, 1.911–3.159), GGT (HR, 1.890, 95% CI, 1.397–2.558), BUN (HR, 1.887, 95% CI, 1.412–2.523), CRE (HR, 1.782, 95% CI, 1.378–2.303), CRP (HR, 1.593, 95% CI, 1.255–2.022), ALP (HR, 1.455, 95% CI, 1.026–2.064), and ALB (HR, 0.587, 95% CI, 0.460–0.750) were associated with OS (Figure 1a).

Multivariate Cox regression identified LDH (HR, 2.024, 95% CI, 1.560–2.627, *p* < 0.001), GGT (HR, 1.875, 95% CI, 1.373–2.562, *p* < 0.001), and ALB (HR, 0.621, 95% CI, 0.479–0.804, *p* < 0.001) as independent prognostic factors for OS in NDMM. These variables were incorporated into the OSS calculation (Figure 1b).

The oxidative stress score formula was derived from the multivariate Cox model in the training cohort: OSS = 0.771 × LDH status + 0.653 × GGT status−0.538 × ALB status, where LDH, GGT, and ALB statuses were each dichotomized based on optimal cutoffs of 232.0 U/L, 16.2 U/L, and 31.2 g/L, respectively. Based on the optimal cutoff in the training cohort, patients with OSS ≥ 0.233 were classified as “high OSS”, and those with OSS < 0.233 were classified as “low OSS”. In the training cohort, 237 patients were assigned to the high OSS group and 537 to the low OSS group. In the validation cohort, 93 patients were classified as high OSS and 240 as low OSS.

### 2.2. Relationship Between OSS and Clinical Characteristics

A comparison of clinical characteristics between different OSS groups is presented in Table 1 and Supplementary Table S1. In both the training and validation cohorts, patients with a high OSS exhibited significantly higher rates of advanced RISS (*p* < 0.001), R2ISS (*p* < 0.001), ISS (*p* < 0.001), elevated β2-Microglobulin (β2-MG) levels (*p* < 0.001), and lower hemoglobin (HGB) levels (*p* < 0.001) compared to those in the low OSS group. In the training cohort, the high OSS group had a higher median age (62 vs. 59 years, *p* = 0.017), and patients in the low OSS group were more likely to undergo autologous transplantation (17.3% vs. 9.7%, *p* = 0.009).

In the validation cohort, a significantly higher prevalence of high-risk cytogenetic abnormalities was observed in the high OSS group compared to the low OSS group (36.2% vs. 18.5%, *p* = 0.028). To further investigate the relationship between OSS and specific cytogenetic features, we analyzed the distribution of four cytogenetic high-risk subtypes across all patients and found that the proportion of 1q21 gain was significantly higher in the high OSS group (*p* < 0.05, Supplementary Figure S1). Despite these baseline differences, treatment distributions were balanced between OSS groups in both cohorts (*p* > 0.05).

### 2.3. Kaplan–Meier Analysis Based on OS and PFS

**Figure 2:**
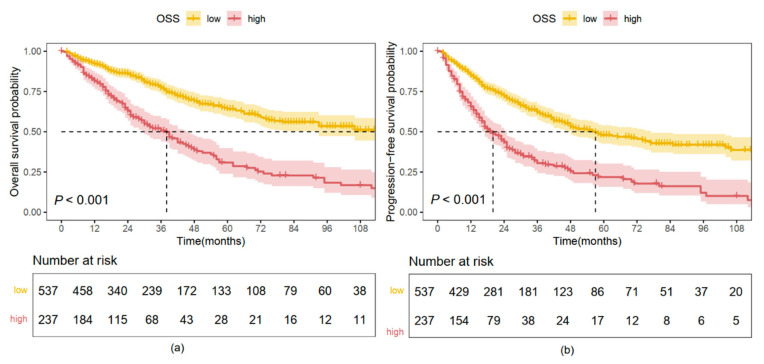
Survival analysis according to OSS stratification in the training cohort. (**a**) Kaplan–Meier overall survival (OS) curves. (**b**) Kaplan–Meier progression-free survival (PFS) curves.

**Figure 3:**
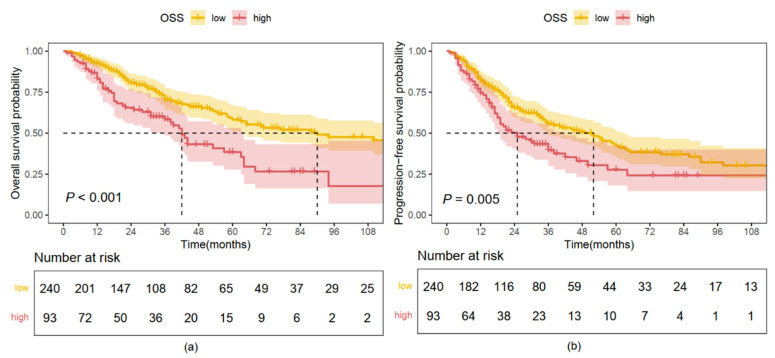
Survival analysis according to OSS stratification in the validation cohort. (**a**) Kaplan–Meier overall survival (OS) curves. (**b**) Kaplan–Meier progression-free survival (PFS) curves.

In the training cohort, the median overall survival (mOS) was 38 months (95% CI, 30–46 months) in the high OSS group, significantly shorter than 138 months (95% CI, 93–NR months) in the low OSS group (HR, 2.680; 95% CI, 2.125–3.380; *p* < 0.001; Figure 2a). Similar findings were observed in the validation cohort, where patients in the high OSS group had an mOS of 42 months (95% CI, 36–64 months), compared to 90 months (95% CI, 63–NR months) in the low OSS group (HR, 2.093; 95% CI, 1.421–3.038; *p* < 0.001; Figure 3a).

For PFS, in the training cohort, the median PFS (mPFS) was 20 months (95% CI, 17–25 months) in the high OSS group, significantly shorter than 57 months (95% CI, 48–77 months) in the low OSS group (HR, 2.441; 95% CI, 1.986–3.000; *p* < 0.001; Figure 2b). In the validation cohort, the mPFS was 25 months (95% CI, 18–42 months) for patients with high OSS, compared to 52 months (95% CI, 36–63 months) in the low OSS group (HR, 1.601; 95% CI, 1.130–2.268; *p* = 0.008; Figure 3b).

### 2.4. Independent Prognostic Significance of OSS

Univariate and multivariate Cox analyses were performed to evaluate the prognostic relevance of OSS and other clinical features for OS and PFS. Univariate analysis demonstrated that high OSS; poor Eastern Cooperative Oncology Group (ECOG) performance status; advanced RISS, R2ISS, and ISS stage; elevated β2-MG; and lower HGB were significantly associated with worse OS in both cohorts (Table 2 and Supplementary Table S2). Similar associations were observed for PFS (Table 3 and Supplementary Table S3).

After adjusting for clinical characteristics and laboratory parameters identified as significant in univariate Cox analysis, multivariate analysis confirmed OSS as an independent prognostic factor for OS (training cohort, HR, 2.038; 95% CI, 1.552–2.677; *p* < 0.001; validation cohort, HR, 1.584; 95% CI, 1.034–2.427; *p* = 0.035) and PFS (training cohort: HR, 1.793; 95% CI, 1.413–2.274; *p* < 0.001; validation cohort: HR, 1.447; 95% CI, 1.017–2.060; *p* = 0.040) in patients with NDMM (Table 2 and Table 3, Supplementary Tables S2 and S3). Notably, OSS remained a significant prognostic marker for OS and PFS across different treatment regimens, including IMiDs, PIs, and IMiDs–PIs-based therapies (Supplementary Figures S2–S4), further supporting its robustness as an independent prognostic indicator.

### 2.5. Model Comparative Assessment and Clinical Benefit Evaluation

**Figure 4:**
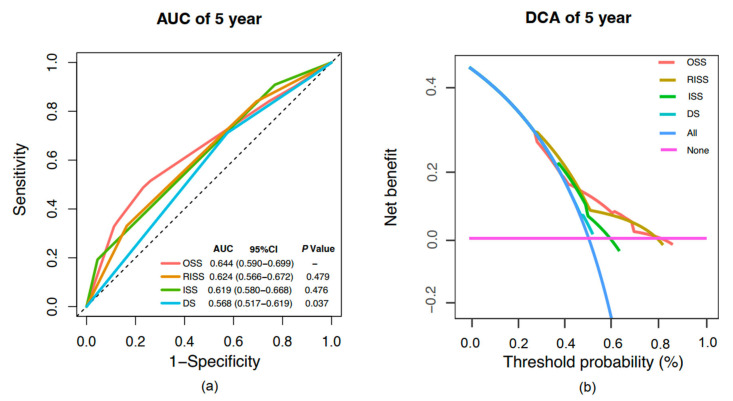
Comparison of prognostic performance between OSS and conventional staging systems. (**a**) Time-dependent ROC (t-ROC) curves for 5-year overall survival comparing the prognostic value of OSS, RISS, ISS, and DS staging systems. (**b**) Decision curve analysis (DCA) assessing the net clinical benefit of each model. Abbreviations: DS, Durie–Salmon staging system; ISS, international staging system; RISS, revised international staging system; OSS, oxidative stress score.

To evaluate the clinical utility of the OSS, we conducted a comparison of its predictive accuracy and clinical benefit with traditional staging systems in patients with complete datasets (Figure 4). The tROC curve analysis revealed that the 5-year AUC values of OSS, RISS, ISS, and DS were 0.644 (95% CI, 0.590–0.699), 0.624 (95% CI, 0.566–0.672), 0.619 (95% CI, 0.580–0.668), and 0.568 (95% CI, 0.517−0.619), respectively. Notably, the OSS demonstrated significantly superior predictive performance compared to the DS system (*p* = 0.037) and exhibited a comparable AUC to both RISS and ISS, suggesting its potential as a simplified surrogate for prognostic stratification (Figure 4a).

The DCA curves demonstrated that OSS, RISS, ISS, and DS provided increased net clinical benefit compared to the “treat-all” and “treat-none” strategies, highlighting their enhanced clinical utility. Within the threshold probability range of 50–80%, OSS and RISS exhibited comparable net benefits, with OSS showing a significantly higher net benefit than DS and ISS in this range (Figure 4b). An additional analysis comparing OSS with R2ISS in the subset of patients with available data for R2ISS evaluation revealed similar 5-year AUCs (0.644 vs. 0.652; *p* = 0.847). Moreover, OSS demonstrated a greater net clinical benefit than R2ISS when threshold probabilities exceeded 45%, while R2ISS performed better below this threshold (Supplementary Figure S5).

**Figure 5:**
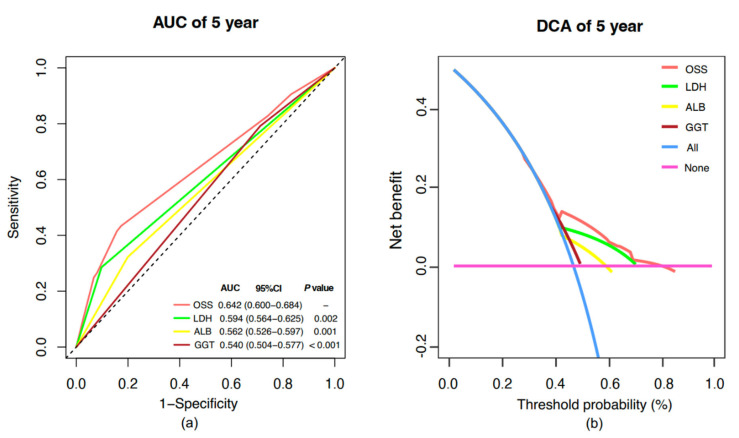
Comparison of prognostic performance between OSS and individual biomarkers. (**a**) Time-dependent ROC (t-ROC) curves for 5-year overall survival comparing the prognostic value of OSS, LDH, ALB, and GGT. (**b**) Decision curve analysis (DCA) assessing the net clinical benefit of each model. Abbreviations: OSS, oxidative stress score; LDH, lactate dehydrogenase; GGT, gamma-glutamyl transferase; ALB, albumin.

Importantly, the OSS model demonstrated significantly superior predictive performance and decision-making utility compared to individual oxidative stress indicators (Figure 5). The 5-year AUC for the OSS model was 0.642 (95% CI, 0.600–0.684), outperforming LDH (AUC = 0.594, 95% CI, 0.564–0.625; *p* = 0.002), ALB (AUC = 0.562, 95% CI, 0.526–0.597; *p* = 0.001), and GGT (AUC = 0.540, 95% CI, 0.504–0.577; *p* < 0.001) (Figure 5a). Furthermore, DCA demonstrated that the OSS model provided a higher net clinical benefit across a wide range of threshold probabilities (42–80%) compared to individual oxidative stress indicators (Figure 5b).

### 2.6. PFS Comparison Among Different Treatment Options

**Figure 6:**
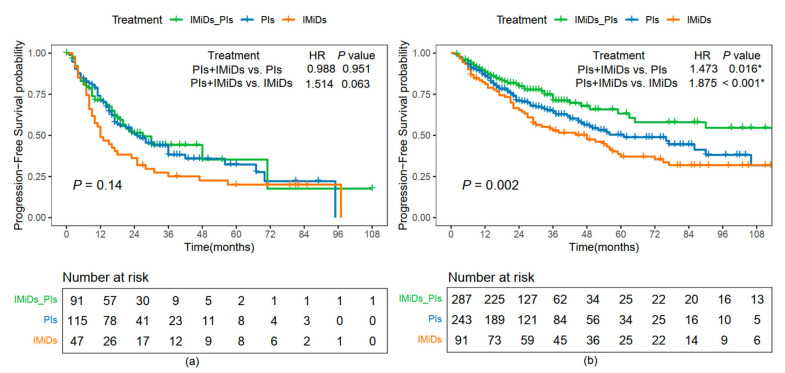
Comparative analysis of PFS based on treatment regimens stratified by OSS. Progression-free survival (PFS) curves for patients with newly diagnosed multiple myeloma (NDMM) receiving different treatment regimens in the OSS-high (**a**) and OSS-low (**b**) groups. Abbreviations: IMiDs, immunomodulatory drugs; PIs, proteasome inhibitors. * indicates *p* < 0.05.

A comparative Kaplan–Meier analysis of PFS was performed in patients with NDMM, stratified by OSS into high-risk (OSS-high) and low-risk (OSS-low) groups across different treatment regimens, to assess the effectiveness of OSS in guiding treatment decisions (Figure 6).

The PFS curves varied according to different chemotherapy regimens, including PIs, IMiDs, and their combination (PIs + IMiDs). In the OSS-high group, even with PIs + IMiDs combination therapy, there was no significant improvement in PFS compared to those treated with PIs or IMiDs alone (HR, 0.988, 95% CI, 0.674–1.448, *p* = 0.951 for PIs and HR, 1.514, 95% CI, 0.978–2.344, *p* = 0.063 for IMiDs; Figure 6a). However, in the OSS-low group, PIs combined with IMiDs demonstrated a significant PFS benefit compared to monotherapy (HR, 1.473, 95% CI, 1.074–2.021, *p* = 0.016 for PIs and HR, 1.875, 95% CI, 1.303–2.697, *p* < 0.001 for IMiDs; Figure 6b).

## 3. Discussion

In our study, we first selected three routinely measured biochemical markers associated with oxidative stress—ALB, LDH, and GGT—to construct a scoring system that reflects the oxidative stress burden in NDMM patients, and we found that it serves as an independent prognostic factor for both OS and PFS. Patients in the high OSS group had significantly worse survival outcomes than those in the low OSS group. Comparisons with traditional staging systems showed that OSS had superior 5-year t-ROC and DCA curves compared to DS staging, with AUC values comparable to ISS and RISS.

Kaplan–Meier analysis revealed distinct PFS patterns between the OSS-high and OSS-low groups. While the combination of proteasome inhibitors and immunomodulatory drugs improved prognosis in low OSS patients, it failed to do so in the high OSS group, suggesting that tumors in patients with high systemic oxidative stress may exhibit reduced responsiveness to standard therapies.

Oxidative stress reactions have been reported to be associated with oncogenesis, tumor progression, and prognosis in multiple tumors. Indeed, due to oxidative stress and intense proliferation, neoplastic plasma cells generate substantial intracellular ROS, causing DNA damage and genomic instability, which may promote chromosomal translocation in MM [23]. Consistently, we observed a higher prevalence of cytogenetic high-risk features in patients with high OSS, particularly 1q21 gain. Collectively, these findings indicate that OSS may serve as a key determinant of prognosis stratification and therapeutic decision-making in MM, potentially accounting for the inferior outcomes observed in NDMM patients with elevated OSS.

LDH is routinely measured in clinical laboratories and has been shown to correlate positively with malondialdehyde, a direct oxidative stress biomarker reflecting lipid peroxidation and oxidative damage [24]. Notably, elevated LDH expression has also been associated with increased ROS production in multiple myeloma cell lines [25]. GGT is closely related to oxidative stress-related risk factors and comorbidities, such as cardiovascular disease, non-alcoholic fatty liver disease, metabolic syndrome, and systemic inflammation [26]. As cells with surface enzyme-regulating glutathione metabolism and the most abundant intracellular antioxidant induced by oxidative stress, tumor cells with high GGT expression demonstrate notable oxidative stress tolerance; therefore, GGT is considered an early and sensitive indicator of oxidative stress in various malignancies [12,27]. ALB, the primary antioxidant in plasma, accounts for over 80% of the free radical capture activity in serum. The detoxification effect of human serum albumin on ROS primarily relies on the activity of glutathione-linked thiol peroxidase [28]. Decreased ALB levels are often linked with poor survival in patients with cancer.

Previous studies have demonstrated that ROS-induced stabilization of hypoxia-inducible factor 1-alpha (HIF-1α) in the bone marrow microenvironment of multiple myeloma patients promotes the expression of proangiogenic genes and contributes to resistance to immunomodulatory drugs and proteasome inhibitors [29]. High levels of systemic oxidative stress may reduce PIs-induced cytotoxicity by enhancing autophagy or activating antioxidant pathways such as the Nrf2 signaling pathway. Activation of Nrf2 has been shown to contribute to the development of PI resistance in MM cells, whereas inhibition of Nrf2 signaling can restore their sensitivity to PIs [9]. These findings suggest that systemic oxidative stress may influence treatment efficacy in NDMM patients through multiple mechanisms.

Interestingly, we observed an association between high OSS and low HGB levels. This aligns with previous studies showing that oxidative stress is linked to eryptosis, a form of programmed red blood cell death [30]. Under oxidative stress, erythrocytes activate Ca^2+^-permeable channels, leading to cell shrinkage and increased membrane sensitivity. This results in the externalization of phosphatidylserine, a hallmark of eryptosis [31]. The development of erythroid cells is susceptible to the build-up of ROS. Hematopoietic stem cells’ ability to perform is compromised by ROS accumulation due to the possibility of DNA damage, which results in changes to hematopoietic stem cell cycling and a loss of quiescence [32].

Our study demonstrates that the OSS, derived from oxidative stress-related markers, is a simple and reliable prognostic stratification system for NDMM, reflecting systemic oxidative stress status and guiding therapeutic decision-making, as validated in a large patient cohort. This integrated approach helps mitigate potential confounding factors by considering multiple oxidative stress-related pathways simultaneously. However, as this was a single-center retrospective study, different treatment strategies and patient populations from specific geographic regions may have introduced biases. In addition, the levels of individual oxidative stress markers could also be influenced by other variables, such as disease burden, comorbidities, or underlying biological characteristics. Therefore, further basic research and prospective studies incorporating additional, more specific oxidative stress markers are needed in the near future to further refine and validate the OSS model and its prognostic relevance in patients with NDMM.

## 4. Materials and Methods

### 4.1. Patient Selection

In this retrospective study, we enrolled 1107 patients with NDMM treated at Sun Yat-sen University Cancer Center (SYSUCC) between March 2001 and August 2023. The inclusion criteria were as follows: (1) age ≥18 years, (2) NDMM based on the International Myeloma Working Group diagnostic criteria, and (3) available pre-treatment biochemical data and sufficient follow-up data for analysis. (4) Patients with other cancer types were excluded from the study.

Ultimately, a total of 1107 patients with NDMM and available pre-treatment clinical and laboratory data were randomly assigned to a training cohort (n = 774) and a validation cohort (n = 333) based on a 7:3 allocation ratio. The study was approved by the SYSUCC Ethics Committee and complied with the Helsinki Declaration and the policies of the Ethics Committee. The institutional review boards granted a waiver of consent for anonymized data collection.

### 4.2. Data Collection

Clinical and laboratory data prior to treatment, including sex, age, Eastern Cooperative Oncology Group (ECOG) status, clinical stage, extramedullary disease, cytogenetic profile, transplantation, treatment, β2-MG (β2-Microglobulin), HGB (hemoglobin), and oxidative stress-related markers (LDH, ALB, CRE, GGT, CRP, ALP, and BUN) were extracted from the medical records database of SYSUCC. Patients were required to fast for 8–12 h before the biochemical analysis.

### 4.3. Outcomes

The primary outcome of this study was progression-free survival (PFS), defined as the time from diagnosis to disease progression, death from any cause, or the last follow-up. The secondary outcome was overall survival (OS), defined as the time from diagnosis to death from any cause or the last follow-up.

### 4.4. Model Construction and OSS Calculation

Oxidative stress indicators were dichotomized into high and low levels using the optimal cutoff value determined by the “surv_cutpoint()” function in the Survminer package, which identifies the threshold that maximizes the log-rank statistic for survival prediction [33]. Univariate Cox regression analysis determined oxidative stress indicators associated with OS, while multivariate Cox regression analysis was used to select significant indicators for OSS model construction in the training cohort. Each oxidative stress indicator was coded as 1 if its value was above the cutoff and 0 otherwise. The corresponding regression coefficients for each oxidative stress indicator were obtained from the multivariate Cox model. The OSS for each patient was calculated using the formula: OSS = sum (corresponding regression coefficient × status of oxidative stress indicator). The optimal cutoff value for OSS was then determined using maximally selected rank statistics, which identify the threshold that maximizes the log-rank statistic for survival prediction. Patients with OSS values above this cutoff were classified as OSS-high, while those below the cutoff were classified as OSS-low.

### 4.5. Statistical Analysis

We retrospectively analyzed the baseline clinical characteristics of patients with NDMM, including the distribution of four cytogenetic high-risk subtypes—del(17p), t(4;14), t(14;16), and 1q21 gain—among patients stratified into high and low OSS groups. Categorical variables were compared using chi-square or Fisher’s exact tests.

Next, we used the Kaplan–Meier method to estimate OS and PFS and compared the differences in survival curves with the log-rank test. Multivariate Cox regression analysis was used to determine independent prognostic factors that showed prognostic significance in univariate Cox analysis among the clinical characteristics and OSS in both the training and validation cohorts, where variables with multicollinearity identified by variance inflation factor (VIF) analysis were excluded.

To further evaluate the clinical utility of the OSS model, we used time-dependent receiver operating characteristic (t-ROC) curves and decision curve analysis (DCA) to compare its performance with individual oxidative stress markers and traditional prognostic models (DS, ISS, and RISS), and with R2ISS in the subset of R2ISS-evaluable patients. All statistical analyses were performed using R version 4.0.3, with statistical significance set at a two-sided *p*-value less than 0.05.

## 5. Conclusions

The OSS model proposed in this study, based on oxidative stress indicators, may serve as a simplified and effective stratification system for evaluating oxidative stress status, predicting prognosis, and guiding therapeutic decision-making in patients with NDMM in a cost-effective, feasible, and minimally invasive manner.

## Figures and Tables

**Table 1:** Clinical characteristics comparison between different oxidative stress score in the training cohort.

Characteristics	OSS-Low (n = 537, %)	OSS-High (n = 237, %)	*p* Value
Sex			0.132
Female	239 (44.5)	91 (38.4)	
Male	298 (55.5)	146 (61.6)	
Age (years)			0.017
Median (IQR)	59 (52–65)	62 (55–68)	
<65	379 (70.6)	146 (61.6)	
≥65	158 (29.4)	91 (38.4)	
ECOG *			0.729
<2	521 (97.7)	228 (97.0)	
≥2	12 (2.3)	7 (3.0)	
DS			<0.001
I	71 (13.2)	14 (5.9)	
II	113 (21.0)	27 (11.4)	
III	353 (65.7)	196 (82.7)	
ISS			<0.001
I	203 (37.8)	29 (12.2)	
II	147 (27.4)	82 (34.6)	
III	187 (34.8)	126 (53.2)	
RISS *			<0.001
I	88 (24.9)	8 (3.9)	
II	246 (69.5)	131 (63.3)	
III	20 (5.6)	68 (32.9)	
R2ISS *			<0.001
I	77 (28.1)	4 (3.0)	
II	76 (27.7)	29 (21.8)	
III	113 (41.2)	80 (60.2)	
IV	8 (2.9)	20 (15.0)	
Extramedullary disease			0.226
No	410 (76.4)	191 (80.6)	
Yes	127 (23.6)	46 (19.4)	
Cytogenetics *			0.543
Standard Risk	203 (74.1)	94 (70.7)	
High Risk	71 (25.9)	39 (29.3)	
Transplant			0.009
No	444 (82.7)	214 (90.3)	
Yes	93 (17.3)	23 (9.7)	
Treatment			0.377
PIs	172 (32.0)	78 (32.9)	
IMiDs	61 (11.4)	33 (13.9)	
IMiDs–PIs	198 (36.9)	72 (30.4)	
Other	106 (19.7)	54 (22.8)	
β2-MG (mg/L)			<0.001
Mean (SD)	5.2 (±7.1)	7.6 (±7.7)	
<3.5	287 (53.4)	55 (23.2)	
≥3.5	250 (46.6)	182 (76.8)	
HGB (g/L)			<0.001
Mean (SD)	106.2 (±26.7)	90.2 (±27.1)	
<120	353 (65.7)	193 (81.4)	
≥120	184 (34.3)	44 (18.6)	

Abbreviations: ECOG, Eastern Cooperative Oncology Group; DS, Durie–Salmon staging system; ISS, international staging system; RISS, revised international staging system; R2ISS, second revision of the international staging system; Pls, proteasome inhibitors; IMiDs, immunomodulatory drugs; β2-MG, β2-microglobulin; HGB, hemoglobin. SD, standard deviation; IQR: interquartile range. High-risk cytogenetic abnormalities include 1q21 gain, del(17p), t(14;16), and t(4;14). * Indicates that data on this variable are not available for some patients.

**Table 2:** The univariate and multivariate Cox regression analyses for OS among the clinical characteristics and OSS in the training cohort.

Variables	Univariate Cox Analysis		Multivariate Cox Analysis	
	HR (95% CI)	*p* Value	HR (95% CI)	*p* Value
Sex				
Female	Reference			
Male	1.158 (0.916–1.465)	0.220		
Age (years)				
<65	Reference		Reference	
≥65	1.480 (1.165–1.880)	0.001	1.077 (0.841–1.379)	0.555
ECOG				
<2	Reference		Reference	
≥2	4.797 (2.735–8.411)	<0.001	3.834 (2.151–6.832)	<0.001
DS				
I	Reference		Reference	
II	1.198 (0.773–1.856)	0.420	1.034 (0.653–1.637)	0.887
III	1.675 (1.149–2.442)	0.007	1.043 (0.680–1.600)	0.847
ISS				
I	Reference			
II	2.042 (1.493–2.791)	<0.001		
III	2.481 (1.830–3.365)	<0.001		
RISS				
I	Reference		Reference	
II	2.515 (1.823–4.813)	<0.001	1.401 (0.802–2.449)	0.236
III	5.711 (3.339–9.768)	<0.001	1.529 (0.781–2.995)	0.216
R2ISS				
I	Reference			
II	1.880 (1.038–3.408)	0.037		
III	2.493 (1.438–4.322)	0.001		
IV	4.280 (2.044–8.964)	<0.001		
Cytogenetic				
Standard Risk	Reference	0.896		
High Risk	1.028 (0.677–1.562)			
Extramedullary Disease				
No	Reference		Reference	
Yes	0.717 (0.527–0.976)	0.035	0.808 (0.584–1.118)	0.198
Transplant				
No	Reference		Reference	
Yes	0.263 (0.157–0.443)	<0.001	0.319 (0.188–0.540)	<0.001
β2-MG (mg/L)				
<3.5	Reference		Reference	
≥3.5	2.198 (1.720–2.809)	<0.001	1.427 (1.056–1.926)	0.020
HGB (g/L)				
<120	Reference		Reference	
≥120	0.532 (0.402–0.704)	<0.001	0.783 (0.565–1.084)	0.141
OSS				
Low	Reference		Reference	
High	2.680 (2.125–3.380)	<0.001	2.038 (1.552–2.677)	<0.001

Abbreviations: ECOG, Eastern Cooperative Oncology Group; DS, Durie–Salmon staging system; ISS, international staging system; RISS, revised international staging system; R2ISS, second revision of the international staging system; β2-MG, β2-microglobulin; HGB, hemoglobin; OSS, oxidative stress score. High-risk cytogenetic abnormalities include 1q21 gain, del(17p), t(14;16), and t(4;14).

**Table 3:** The univariate and multivariate Cox regression analyses for PFS among the clinical characteristics and OSS in the training cohort.

Variables	Univariate Cox Analysis		Multivariate Cox Analysis	
	HR (95% CI)	*p* Value	HR (95% CI)	*p* Value
Sex				
Female	Reference			
Male	1.070 (0.871–1.314)	0.519		
Age (year)				
<65	Reference		Reference	
≥65	1.306 (1.045–1.613)	0.013	0.980 (0.786–1.221)	0.855
ECOG				
<2	Reference		Reference	
≥2	3.068 (1.759–5.352)	<0.001	2.608 (1.486–4.575)	0.001
DS				
I	Reference			
II	1.411 (0.949–2.098)	0.089		
III	1.839 (1.302–2.598)	0.001		
ISS				
I	Reference			
II	1.672 (1.275–2.192)	<0.001		
III	2.027 (1.563–2.2629)	<0.001		
RISS				
I	Reference		Reference	
II	1.788 (1.214–2.632)	0.003	1.140 (0.738–1.761)	0.554
III	4.181 (2.690–6.498)	<0.001	1.708 (0.999–2.920)	0.051
R2ISS				
I	Reference			
II	1.517 (0.949–2.424)	0.082		
III	1.797 (1.169–2.764)	0.008		
IV	4.020 (2.233–7.237)	<0.001		
Extramedullary Disease				
No	Reference			
Yes	0.874 (0.678–1.126)	0.296		
Cytogenetic				
Standard Risk	Reference			
High Risk	1.092 (0.777–1.536)	0.611		
Transplant				
No	Reference		Reference	
Yes	0.466 (0.326–0.667)	<0.001	0.537 (0.372–0.775)	0.001
β2-MG (mg/L)				
<3.5	Reference		Reference	
≥3.5	1.797 (1.455–2.219)	<0.001	1.231 (0.956–1.568)	0.107
HGB (g/L)				
<120	Reference		Reference	
≥120	0.550 (0.432–0.699)	<0.001	0.680 (0.522–0.887)	0.004
OSS				
Low	Reference		Reference	
High	2.441 (1.986–3.000)	<0.001	1.793 (1.413–2.274)	<0.001

Abbreviations: ECOG, Eastern Cooperative Oncology Group; DS, Durie–Salmon staging system; ISS, international staging system; RISS, revised international staging system; R2ISS, second revision of the international staging system; β2-MG, β2-microglobulin; HGB, hemoglobin; OSS, oxidative stress score. High-risk cytogenetic abnormalities include 1q21 gain, del(17p), t(14;16), and t(4;14).

## Data Availability

These data are available by individual application to the corresponding authors.

## Supplementary Materials

The following supporting information can be downloaded at: https://www.mdpi.com/article/10.3390/ph18060878/s1, Table S1: Clinical characteristics comparison between different oxidative stress score in the validation cohort; Table S2: The univariate and multivariate Cox regression analyses for OS among the clinical characteristics and OSS in the validation cohort; Table S3: The univariate and multivariate Cox regression analyses for PFS among the clinical characteristics and OSS in the validation cohort; Figure S1: Distribution of Four Cytogenetic High-Risk Subtypes Among Patients; Figure S2: Kaplan–Meier curves for PFS in patients receiving IMiD–PI–based therapy stratified by OSS; Figure S3: Kaplan–Meier curves for PFS in patients receiving IMiD-based therapy stratified by OSS; Figure S4: Kaplan–Meier curves for PFS in patients receiving PI-based therapy stratified by OSS; Figure S5: Comparison of prognostic performance between OSS and R2ISS.
