# Supplementary material for: Oxidative Stress Score as a Simplified Surrogate for Prognostic Stratification and Therapeutic Decision-Making in Multiple Myeloma
Source: Pharmaceuticals (Basel). 2025 Jun 12;18(6):878. doi: 10.3390/ph18060878 (PMC12195873; doi:10.3390/ph18060878)

**Table S1:** Clinical characteristics comparison between different oxidative stress score in the validation cohort.

| **Characteristics** | **OSS-low (n = 240, %)** | **OSS-high (n = 93, %)** | ***P* value** |
|---|---|---|---|
| Sex (%) | | | 0.110 |
| Female | 94 (39.2) | 27 (29.0) | |
| Male | 146 (60.8) | 66 (71.0) | |
| Age (%) | | | 0.214 |
| Median (IQR) | 60 (53-66) | 61.0 (56-67) | |
| < 65 | 166 (69.2) | 57 (61.3) | |
| ≥ 65 | 74 (30.8) | 36 (38.7) | |
| ECOG (%) | | | 0.127 |
| <2 | 235 (98.3) | 86 (94.5) | |
| ≥2 | 4 (1.7) | 5 (5.5) | |
| DS (%) | | | 0.057 |
| I | 29 (12.1) | 5 (5.4) | |
| II | 56 (23.3) | 16 (17.2) | |
| III | 155 (64.6) | 72 (77.4) | |
| ISS (%) | | | < 0.001 |
| I | 94 (39.2) | 14 (15.1) | |
| II | 73 (30.4) | 30 (32.3) | |
| III | 73 (30.4) | 49 (52.7) | |
| RISS (%) | | | < 0.001 |
| I | 44 (27.7) | 2 (2.5) | |
| II | 110 (69.2) | 55 (68.8) | |
| III | 5 (3.1) | 23 (28.7) | |
| R2ISS (%) | | | < 0.001 |
| I | 41 (33.3) | 1 (2.1) | |
| II | 35 (28.5) | 4 (8.5) | |
| III | 46 (37.4) | 35 (74.5) | |
| IV | 1 (0.8) | 7 (14.9) | |
| Extramedullary (%) | | | 0.765 |
| No | 175 (72.9) | 70 (75.3) | |
| Yes | 65 (27.1) | 23 (24.7) | |
| Cytogenetic | | | |
| Standard Risk | 100 (81.3) | 30 (63.8) | 0.028 |
| High Risk | 23 (18.5) | 17 (36.2) | |
| Transplant (%) | | | 0.272 |
| No | 201 (83.8) | 83 (89.2) | |
| Yes | 39 (16.2) | 10 (10.8) | |

| Characteristics | OSS-low (n = 240, %) | OSS-high (n = 93, %) | *P* value |
|---|---|---|---|
| Treatment (%) | | | 0.092 |
| PIs | 71 (29.6) | 35 (37.6) | |
| IMiDs | 30 (12.5) | 14 (15.1) | |
| IMiDs_PIs | 89 (37.1) | 21 (22.6) | |
| Others | 50 (20.8)) | 23 (24.7) | |
| β2-MG (%) | | | < 0.001 |
| Mean (SD) | 4.84 (7.5) | 8.88 (10.6) | |
| < 3.5 | 134 (55.8) | 27 (29.0) | |
| ≥ 3.5 | 106 (44.2) | 66 (71.0) | |
| HGB (%) | | | < 0.001 |
| Mean (SD) | | | |
| < 120 | 146 (60.8) | 81 (87.1) | |
| ≥ 120 | 94 (39.2) | 12 (12.9) | |

Abbreviations: ECOG, Eastern Cooperative Oncology Group; DS, Durie-Salmon staging system; ISS, international staging system; RISS, revised international staging system; R2ISS, second revision of the international staging system; Pls, proteasome inhibitors; IMiDs, immunomodulatory drugs; β2-MG, β2-microglobulin; HGB, hemoglobin. SD, standard deviation; IQR: interquartile range. All P-values are based on comparisons between categorical variables.

**Table S2.** The univariate and multivariate Cox regression analyses for OS among the clinical characteristics and OSS in the validation cohort.

| Variables | Uni-variate Cox analysis | | Multi-variate Cox analysis | |
|---|---|---|---|---|
| | **HR(95%CI)** | ***P* value** | **HR(95%CI)** | ***P* value** |
| Sex | | | | |
| Female | Reference | | | |
| Male | 0.819 (0.578-1.162) | 0.263 | | |
| Age (year) | | | | |
| < 65 | Reference | | | |
| ≥ 65 | 1.099 (0.764-1.583) | 0.610 | | |
| ECOG | | | | |
| < 2 | Reference | | Reference | |
| ≥ 2 | 3.714 (1.728-7.981) | **0.001** | 4.633 (2.082-10.309) | < 0.001 |
| DS | | | | |
| I | Reference | | | |
| II | 1.062 (0.553-2.040) | 0.856 | | |
| III | 1.197 (1.050-2.371) | 0.546 | | |
| ISS | | | | |
| I | Reference | | Reference | |
| II | 2.163 (1.377-3.398) | **0.001** | 1.142 (0.636-2.051) | 0.496 |
| III | 2.536 (1.599-4.020) | **< 0.001** | 1.600 (0.847-3.022) | 0.311 |

| Variables | Uni-variate Cox analysis | | Multi-variate Cox analysis | |
|---|---|---|---|---|
| | HR(95%CI) | *P* value | HR(95%CI) | *P* value |
| RISS | | | | |
| I | Reference | | Reference | |
| II | 3.151 (1.519-6.537) | 0.002 | 1.832 (0.739-4.542) | **0.191** |
| III | 8.394 (3.491-20.182) | < **0.001** | 3.018 (1.020-8.928) | **0.046** |
| R2ISS | | | | |
| I | Reference | | | |
| II | 2.090 (0.956-4.572) | 0.065 | | |
| III | 2.492 (1.211-5.126) | **0.013** | | |
| IV | 3.501 (0.951-12.886) | 0.060 | | |
| Extramedullary disease | | | | |
| No | Reference | | | |
| Yes | 1.035 (0.688-1.557) | 0.869 | | |
| Transplant | | | | |
| No | Reference | | | |
| Yes | 0.832 (0.500-1.385) | 0.479 | | |
| Cytogenetic | | | | |
| Standard Risk | Reference | | | |
| High Risk | 1.225 (0.672-2.233) | 0.507 | | |
| β2-MG (mg/L) | | | | |
| < 3.5 | Reference | | Reference | |
| ≥ 3.5 | 1.879 (1.322-2.672) | < **0.001** | 0.976 (0.595-1.600) | 0.922 |
| HGB (g/L) | | | | |
| < 120 | Reference | | Reference | |
| ≥ 120 | 0.483 (0.319-0.731) | **0.001** | 0.668 (0.414-1.077) | 0.098 |
| OSS | | | | |
| Low | Reference | | Reference | |
| High | 2.093 (1.421-3.083) | < **0.001** | 1.584 (1.034-2.427) | **0.035** |

Abbreviations: ECOG, Eastern Cooperative Oncology Group; DS, Durie-Salmon staging system; ISS, international staging system; RISS, revised international staging system; R2ISS, second revision of the international staging system; Pls, proteasome inhibitors; IMiDs, immunomodulatory drugs; β2-MG, β2-microglobulin; HGB, hemoglobin. OSS, oxidative stress score.

**Table S3.** The univariate and multivariate Cox regression analyses for PFS among the clinical characteristics and OSS in the validation cohort.

| **Variables** | **Uni-variate Cox analysis** | | **Multi-variate Cox analysis** | |
|---|---|---|---|---|
| | **HR (95%CI)** | ***P* value** | **HR (95%CI)** | ***P* value** |
| Sex | | | | |
| Male | Reference | | | |
| Female | 0.919 (0.674-1.255) | 0.597 | | |
| Age (year) | | | | |
| < 65 | Reference | | | |
| ≥ 65 | 1.033 (0.747-1.427) | 0.846 | | |
| ECOG | | | | |
| < 2 | Reference | | Reference | |
| ≥ 2 | 2.476 (1.157-5.296) | **0.019** | 3.430 (1.548-7.600) | **0.002** |
| DS | | | | |
| I | Reference | | | |
| II | 1.258 (0.701-2.261) | 0.442 | | |
| III | 1.461 (0.864-2.470) | 0.157 | | |
| ISS | | | | |
| I | Reference | | Reference | |
| II | 1.875 (1.279-2.747) | 0.001 | 1.378 (0.838-2.266) | 0.207 |
| III | 1.651 (1.114-2.449) | **0.013** | 1.598 (0.940-2.717) | 0.083 |
| RISS | | | | |
| I | Reference | | | |
| II | 2.055 (1.208-3.498) | 0.008 | | |
| III | 3.720 (1.865-7.420) | **< 0.001** | | |
| R2ISS | | | | |
| I | Reference | | | |
| II | 1.966 (1.056-3.662) | **0.033** | | |
| III | 1.423 (0.797-2.540) | 0.233 | | |
| IV | 7.367 (2.794-19.428) | **< 0.001** | | |
| Extramedullary disease | | | | |
| No | Reference | | | |
| Yes | 0.965 (0.675-1.382) | 0.847 | | |
| Transplant | | | | |
| No | Reference | | | |
| Yes | 1.013 (0.661-1.552) | 0.954 | | |
| Cytogenetic | | | | |
| Standard Risk | Reference | | Reference | |
| High Risk | 1.785 (1.094-2.912) | **0.020** | 1.878 (1.145-3.081) | **0.013** |
| β2-MG (mg/L) | | | | |
| < 3.5 | Reference | | Reference | |
| ≥ 3.5 | 1.426 (1.050-1.937) | **0.023** | 0.765 (0.488-1.199) | **0.242** |

| Variables | Uni-variate Cox analysis | | Multi-variate Cox analysis | |
|---|---|---|---|---|
| | HR (95%CI) | *P* value | HR (95%CI) | *P* value |
| HGB (g/L) | | | | |
| < 120 | Reference | | Reference | |
| ≥ 120 | 0.554 (0.391-0.785) | **0.001** | 0.628 (0.420-0.940) | **0.024** |
| OSS | | | | |
| Low | Reference | | Reference | |
| High | 1.601 (1.130-2.268) | **0.008** | 1.447 (1.017-2.060) | **0.040** |

Abbreviations: ECOG, Eastern Cooperative Oncology Group; DS, Durie-Salmon staging system; ISS, international staging system; RISS, revised international staging system; R2ISS, second revision of the international staging system; Pls, proteasome inhibitors; IMiDs, immunomodulatory drugs; β2-MG, β2-microglobulin; HGB, hemoglobin. OSS, oxidative stress score.

**Figure S1.** Distribution of Four Cytogenetic High-Risk Subtypes Among Patients

The frequencies of del(17p), t(4;14), t(14;16), and 1q21 gain are shown for patients stratified by oxidative stress score (OSS) into high and low groups.

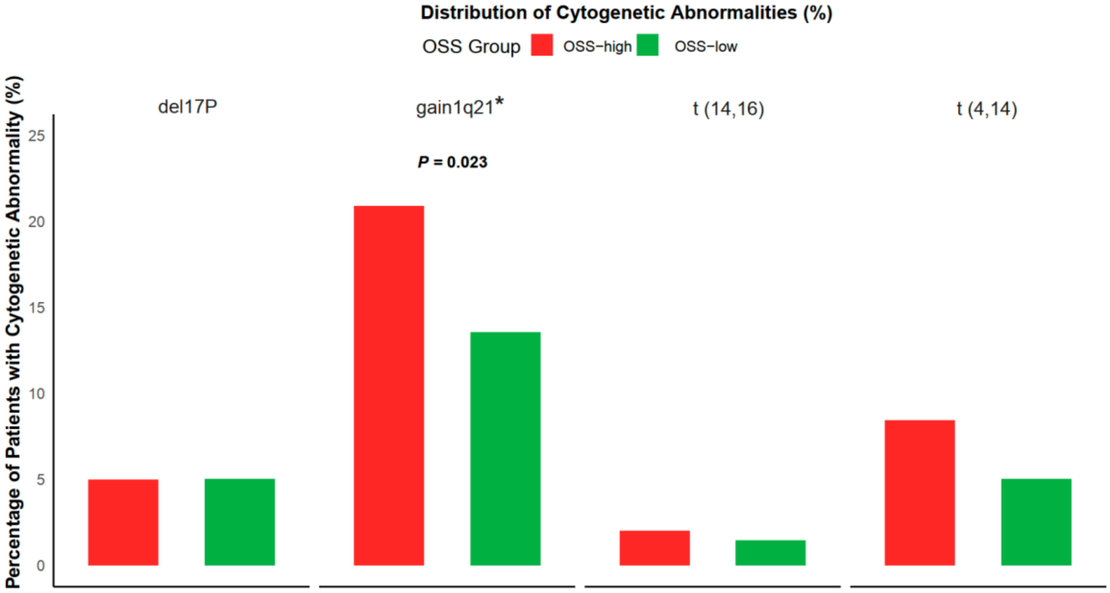

**Figure S2.** Kaplan–Meier curves for PFS in patients receiving IMiD–PI–based therapy stratified by OSS. Progression-free survival (PFS) comparison between high and low OSS groups among patients treated with combined immunomodulatory drugs (IMiDs) and proteasome inhibitors (PIs).

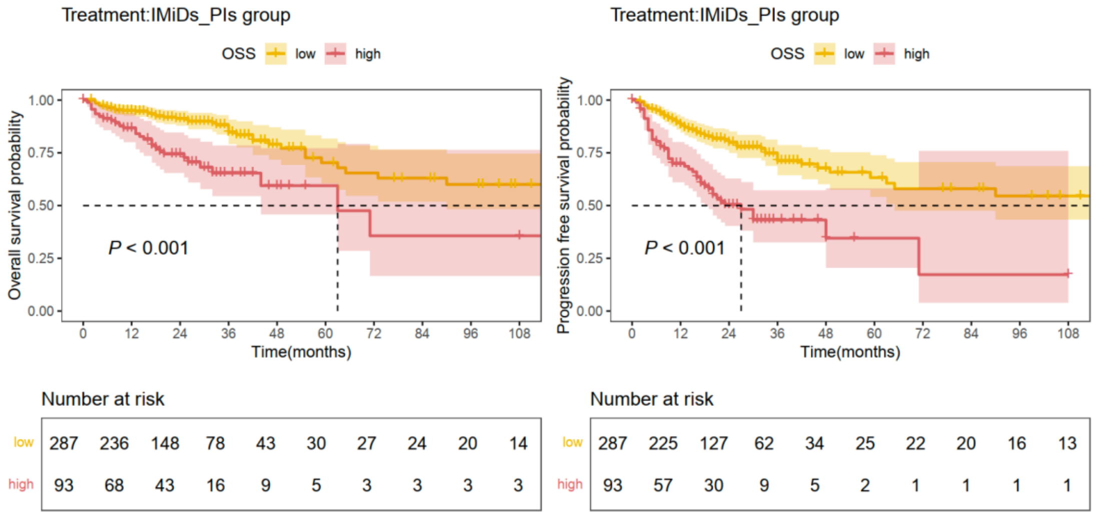


**Figure S3.** Kaplan–Meier curves for PFS in patients receiving IMiD-based therapy stratified by OSS. Progression-free survival (PFS) comparison between high and low OSS groups among patients treated with immunomodulatory drug (IMiD) monotherapy.

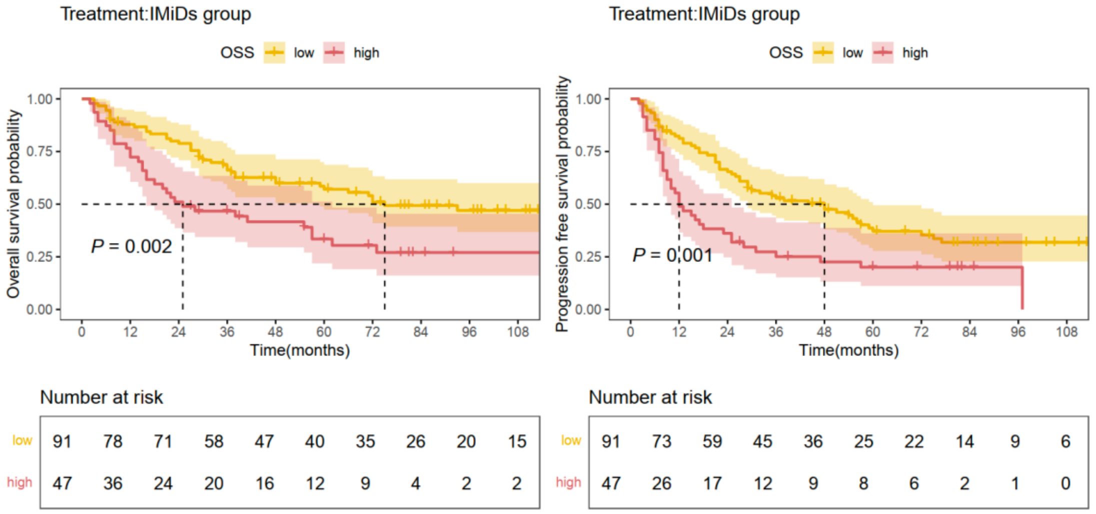

**Figure S4.** Kaplan–Meier curves for PFS in patients receiving PI-based therapy stratified by OSS. Progression-free survival (PFS) comparison between high and low OSS groups among patients treated with proteasome inhibitor (PI) monotherapy.

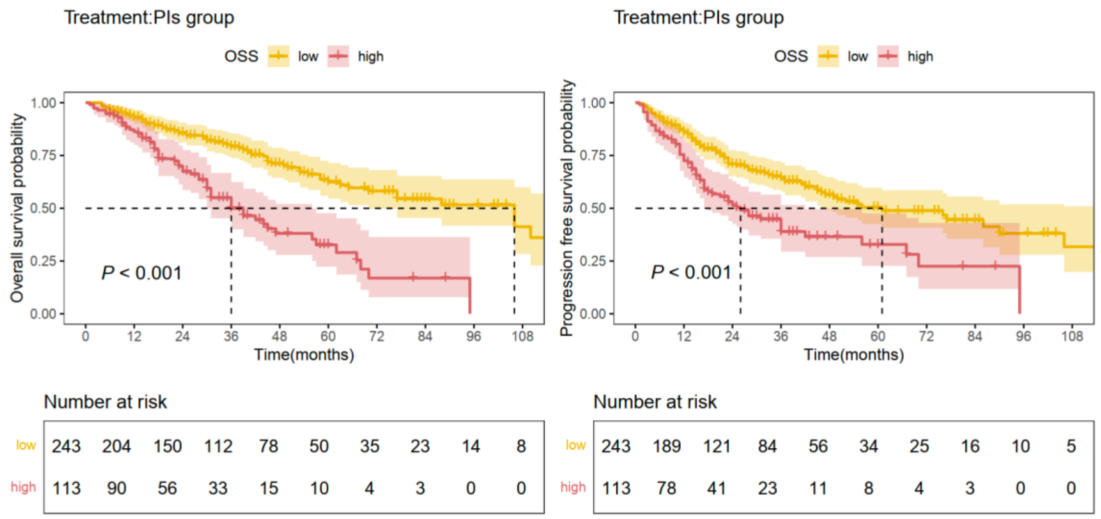


**Figure S5.** Comparison of prognostic performance between OSS and R2ISS**.** Time-dependent ROC (t-ROC) and decision curve analysis (DCA) were used to compare the 5-year overall survival (OS) predictive value and decision-making utility of the oxidative stress score (OSS) and second revision of the international staging system (R2ISS).

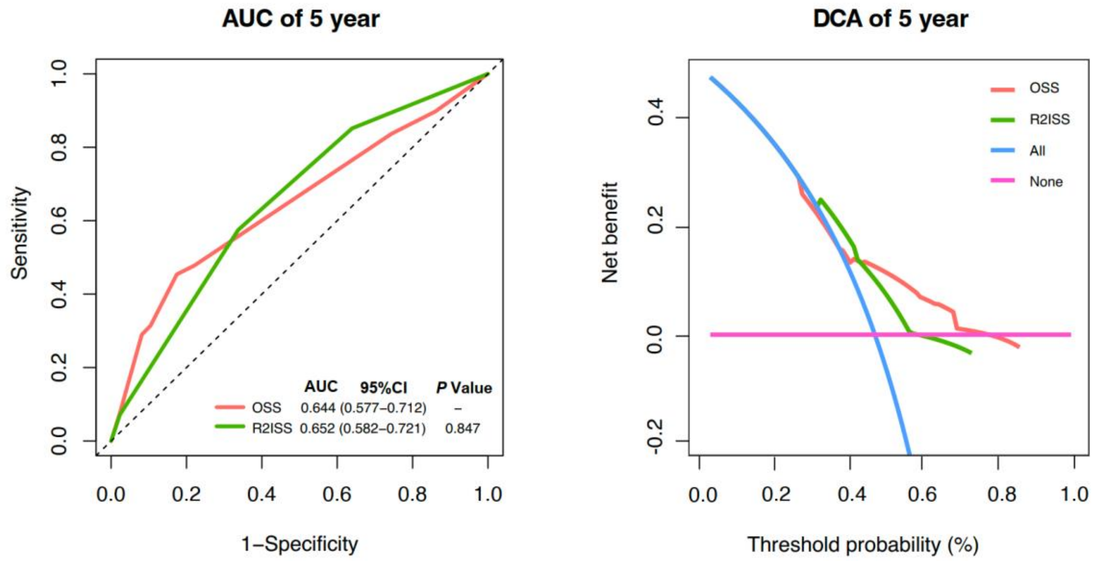

Supplement: Supplementary file 1 [file pharmaceuticals-18-00878-s001.zip › pharmaceuticals-3626358-supplementary.pdf]
